# GBSX: a toolkit for experimental design and demultiplexing genotyping by sequencing experiments

**DOI:** 10.1186/s12859-015-0514-3

**Published:** 2015-03-06

**Authors:** Koen Herten, Matthew S Hestand, Joris R Vermeesch, Jeroen KJ Van Houdt

**Affiliations:** Center for Human Genetics, KU Leuven, Herestraat 49, Leuven, 3000 Belgium; Genomics Core, UZ Leuven, Herestraat 49, Leuven, 3000 Belgium

**Keywords:** GBS, In-line barcodes, Demultiplexing

## Abstract

**Background:**

Massive parallel sequencing is a powerful tool for variant discovery and genotyping. To reduce costs, sequencing of restriction enzyme based reduced representation libraries can be utilized. This technology is generally referred to as Genotyping By Sequencing (GBS). To deal with GBS experimental design and initial processing specific bioinformatic tools are needed.

**Results:**

GBSX is a package that assists in selecting the appropriate enzyme and the design of compatible in-line barcodes. Post sequencing, it performs optimized demultiplexing using these barcodes to create fastq files per barcode which can easily be plugged into existing variant analysis pipelines. Here we demonstrate the usability of the GBSX toolkit and demonstrate improved in-line barcode demultiplexing and trimming performance compared to existing tools.

**Conclusions:**

GBSX provides an easy to use suite of tools for designing and demultiplexing of GBS experiments.

## Background

Next generation sequencing (NGS) enables whole genome sequencing or targeted sequencing of a large fraction of the genome. Restriction enzyme digestion can be used for reducing the complexity of the genome by a reproducible selection of genomic regions. In combination with NGS this has resulted in different strategies for cost effective genome-wide marker discovery and genotyping [1]. The genotyping by sequencing (GBS) strategy, as developed by Elshire et al. [2], uses a single restriction enzyme for the digestion of genomic DNA, followed by the ligation of adaptors that allow PCR amplification and subsequent sequencing on an NGS platform. The Illumina machines are currently the most used NGS platforms. During this procedure fragments of an amplifiable length are selected and the complexity of the target is reduced (a reduced representation library). This results in a reproducible selection of genomic DNA fragments. To date, this cost-reduction method has been used for genotyping a variety of species, including maize, barley, soybeans, oats, watermelon, and cattle [2-6].

**Figure 1:**
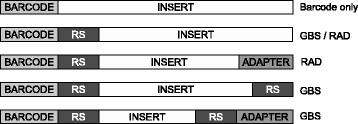
**Library types with in-line barcode.** The GBSX demultiplexer can handle barcode only sequences, sequences starting with barcode and restriction enzyme site (RS), and those that also end with RS and/or common adapter.

However, the restriction based reduction introduces low-diversity in the initial bases of the sequencing library due to every sequence starting with an identical restriction digest recognition sequence. This interferes with (Illumina) cluster identification, resulting in a significant loss of data [7-10]. A solution to this problem is the addition of in-line barcodes (Figure 1) with different lengths. The length difference ensures that the restriction digest recognition sequence, present in all library fragments, is not read in the same sequencing cycle.

Illumina uses barcodes (indices) that reside in the adaptor and not in the sequence read. Hence, Illumina’s demultiplexing software does not natively support in-line barcodes for sample identification. The Tassel software [2] provides a pipeline for analyzing single read GBS data, but has some restrictions. Tassel 3.0 evaluates reads for the presence of an expected barcode, without the presence of restriction enzyme cut sites. If a barcode is found, the barcode is trimmed from the read, which is further trimmed to 64 bases, retaining the enzyme cut site. When a second cut site is found, a polyA tail replaces the remaining sequence. The output of this demultiplexing step is a set of binary files that can only be used within the Tassel framework, as they cannot be converted to standard fastq or fasta formats. An alternative analysis strategy for GBS data is to process them as Restriction site Associated DNA (RAD) data. Although the RAD and GBS protocol are very similar the resulting data have distinct properties (while maintaining sample information). GBS fragments will always start and end with a restriction site whereas RAD fragments will only have a single restriction site sequence. Furthermore, GBS sequences for a certain restriction site will always start and end at the same position whereas this is variable for RAD sequences. The Stacks [11] package can handle RAD data, including the ability to input paired-end fastq files and output fastq files per sample via their demultiplex tools (module process_radtags and process_shortreads). However, Stacks currently can not handle barcodes of different lengths and does not utilize the restriction site for trimming. Byrne *et al.* [12] have used an adjusted Stacks pipeline to analyze GBS data that utilizes Sabre for demultiplexing. Reads are checked for adaptors and, if found, the reads are removed. Otherwise, reads are trimmed to a length of 64 basepairs (similar to the Tassel pipeline) and Stacks is used to complete the analysis. Therefore, this pipeline can handle barcodes of different lengths, but it has limited trimming capabilities. Theoretically, the hard cut-off of 64 basepairs could be avoided and trimming improved by incorporating more dedicated trimming modules into these pipelines, such as the fastx-toolkit [13] or trimmomatic [14]. To improve upon current GBS pipelines we set out to develop a pipeline that would provide easy-to-incorporate text-format output using: 1) more sensitive algorithms for demultiplexing and 2) a more sophisticated trimming method that searches for and trims on the restriction site containing adaptor sequence, as opposed to throwing out adaptor containing reads, hard-trimming to 64 bases, or ignoring the restriction site during trimming.

Here we present a suite of tools that can aid in the design of GBS experiments and facilitates the initial processing. It provides the following tools:

- an *in silico* digest for evaluating restriction enzymes
- in-line barcode design, given a selected restriction enzyme
- a sequencing demultiplexer based on in-line barcodes
- supplemental tools for data simulation and post-sequencing barcode discovery

## Implementation

GBSX is developed as an easy to use toolkit for both users and developers. The *in silico* digest script is written in Perl and requires Bioperl modules [15]. All other tools are part of a single Java program. For memory reasons BioJava is not used for handling fastq or fastq.gz input and output files. Instead a buffered reader/writer is used. All scripts are licensed under GNU General Public License (GPL) version 3.

### In silico digest

The GBSX digest script performs an *in silico* digest of a reference genome in fasta format utilizing a user provided enzyme restriction digest sequence. This is done by using BioPerl modules to identify all restriction cut site positions. Then, based on an adjustable fragment size range and read length parameters, report statistics are calculated on the number and distribution of sequencable fragments. A bed format file is also generated of predicted sequenced bases, which can be viewed in genome browsers or used for annotation, such as overlap with known variants or repetitive elements. The same analysis can also be performed using two enzyme restriction sites, with statistics provided on the joint and separate cut sites. If requested, the number of sequencable fragments containing a third digest site can also be reported.

### Barcode design

**Figure 2:**
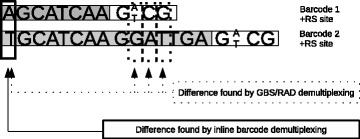
**The importance of a good barcode design.** This image shows 2 barcodes, completed with the restriction enzyme recognition site of ApeKI. If these barcodes are used for the demultiplexing of GBS or RAD data with the ApeKI enzyme, the software will recognize the correct barcodes (sample) because of the Hamming distance between the barcodes. When the barcodes are used with another or no enzyme, the barcodes will have a different distance. This could result in misdemultiplexing of the reads.

The barcode generator designs random self-correcting barcodes based on Hamming codes as described in Bystrykh *et al.* [16]. Generated barcodes vary in length and have an equal representation of the different nucleotides at every position in order to mitigate possible problems due to a low diversity of the library. The algorithm creates a random barcode set given the restriction enzyme of choice and the desired number of barcodes. Our implementation uses Hamming (15,11) codes (11 data bits and 4 parity bits). During the design process shorter barcodes are extended with a polyA sequence in order to be compliant with the Hamming code. For the experimental use of the barcodes the polyA sequences are removed. As such the self-correcting nature of the Hamming code is retained. The barcodes differ by at least a Hamming distance of three and up to one substitution error can be corrected. Additional constraints are that barcodes cannot contain restriction enzyme recognition sites and that shorter barcodes cannot be identical to a partial sequence of a longer barcode. The combination of the smallest barcode and restriction enzyme recognition site must have a Hamming distance of 3 or more compared to the start of all other barcodes and restriction enzyme recognition sites. Figure 2 illustrates two barcodes designed with a restriction site. Due to the design constraints these two barcodes have a Hamming distance of 4. If the same barcodes would be used without the enzyme, or without the constraints, both barcodes will be demultiplexed as being the shortest barcode (Hamming distance of 1). Using another restriction enzyme could introduce a smaller Hamming distance, resulting in the same misassignment. Hence, for optimal usage, the designed barcodes can only be used in combination with the corresponding restriction enzyme. Using different or no restriction enzyme in combination with these barcodes will result in incorrect demultiplexing, and hence incorrect results (Figure 2).

### GBSX demultiplexer

The GBSX demultiplexer is capable of demultiplexing fastq files with a variety of in-line barcode format sequences (Figure 1), including:

- in-line barcode sequences without a restriction enzyme cutsite at the start of each read
- RAD sequences: in-line barcode and restriction enzyme cutsite at the start of each read and an optional adaptor at the read end
- GBS sequences: in-line barcode and restriction enzyme cutsite at the start of each read and an optional restriction enzyme cutsite and adaptor at the read end

The GBSX demultiplexer requires one (for single-end reads) or two (for paired-end reads) fastq files and a parameters file as input. The fastq files can be gziped. The parameters file is a tab-delimited text file with the sample names and their associated barcode and enzyme. The start of the fastq sequence is checked for the barcodes provided in the parameters file. The barcode and enzyme (if any) are trimmed from the sequence and corresponding quality values. The end of the sequence is then trimmed for a possible enzyme cut site followed by the start of the common adapter (optional) (Figure 1). Demultiplex statistics are generated, including the number of mismatches in the barcodes, percentage of the sample in the data, number of basepairs, and overall quality.

Since GBS often uses short insert sizes read 1 and read 2 of read pairs, when using paired-end sequencing, often overlap. Therefore we include an additional analysis step for paired-end read trimming to include a consistency check across both reads. Read 1 is trimmed as described earlier and the read 2 sequence is trimmed for the enzyme cut site followed by the complement of the barcode. Trimmed read 1 and read 2 sequences are then assessed if adaptor trimming was performed similarly in both reads. Trimming is corrected in case of any inconsistency. There is an inconsistency when one or both reads are trimmed, but both reads do not have the same length. In this case, the start of the longest read has to be the complement of the end of the shortest read. This is checked for the restriction enzyme cutsite and the first bases of the longest read (number of bases variates as the length of the longest barcode, with the allowed mismatch). When there is a match, the longest read is trimmed to the length of the shortest read. When this does not match, the trimming of the longest read is assumed to be correct. The shortest read is corrected by trimming the original read.

### Barcode discovery

The barcode discovery tool counts all possible barcodes (with length between 6 and 16 by default) in a fastq file. This can be used when a large portion of the demultiplex is undetermined. The barcode discovery tool can also be used to identify unexpected barcodes, or global sequencing errors. This tool utilizes a standard restriction enzyme annotation file, based on the Stacks standard enzymes. The annotation file can be replaced by a new file or extended by the user. The output of this tool are tab-delimited files. One file is created per barcode length. In these files are the following columns: the barcode sequence, the name of the restriction enzyme associated with the barcode, and the number of reads identified with the barcode.

### GBSX simulator

The GBSX Simulator tool was developed for evaluating the performance of the GBSX toolkit by generating simulated GBS data with sequencing errors. The simulator uses a fasta file and a barcode file as input. The fasta file contains sequences of different lengths, all starting and ending with the restriction enzyme recognition site. The barcode file contains all barcodes that need to be included in the simulation.

For every fasta sequence, reads are simulated of the specified length completed with the common adapter (if necessary). The reads per locus parameter determines the total number of reads that will be generated per sample. An equal number of reads is generated from both ends of the fasta sequence. The tool supports the generation of both single-end and paired-end reads. While generating the reads errors are introduced under the simple assumption that sequencing will generate a single error every 100 bases. The error base is changed to another random base (including N). The simulation generates statistics containing the number of correct barcodes and barcodes with one mismatch.

## Results and discussion

GBSX is a toolkit developed to enhance GBS experimental design and analysis. To compare demultiplexing and trimming to existing tools we simulated data based on chromosome 21 of the human reference genome (hg19). The chromosome 21 fasta file was used as input for the *in silico* digest script using the restriction enzyme ApeKI, sequence GˆCWGC. The additional non-default parameters used were minimum fragment size of 60bp and maximum fragment size of 200 bp. The resulting bed file was adjusted so all fragments include the complete restriction cutsite (here 3 bases where added to account for overhangs). This bed file was used, together with the fasta file of the reference, as input for the bedtools bedtofasta toolkit [17]. This resulted in a fasta file with all target restriction fragment sequences. This fasta file was then used as input for the GBSX Simulator. Ten experimental data sets of paired-end reads were generated. Each data set generated used different barcodes targeting a different number of samples (between 5-20). The barcodes were generated per experiment with the Barcode Generator with a variable length between 8 and 16. In addition, the simulated data sets had varying synthetic read depths for restriction targeted loci (between 2 and 10 reads). The simulation was done twice for each experimental group, either including or not including the ApeKI restriction enzyme sequence. For single end synthetic data the first read file of paired-end reads was used.

GBSX and Stacks, tools which can both demultiplex and trim simultaneosly, were evaluated with these datasets. In addition we evaluated the demultiplexing of Sabre, which is part of a published GBS pipeline [12].

Demultiplexing was evaluated with the simulated data using three different tools: GBSX, Stacks, and Sabre. The datasets with ApeKI restriction enzyme sequence were demultiplexed with GBSX (GBS and RAD demultiplexers) and Stacks (process_radtags). The datasets without restriction enzyme sequence were demultiplexed with GBSX (as an in-line barcode demultiplexer with adapter removal, no restriction enzyme recognition sites), Stacks (process_shortreads), and Sabre. For all tools 1 mismatch was permitted in the barcode and 1 mismatch was permitted in the restriction enzyme recognition site (if applicable). For GBSX and Stacks tools the common adapter (with a length of 14 bp) was supplied. Tassel was not included in this performance analysis since its demultiplexing results in a binary file, of which no tools are available to convert to a text format file (e.g. fasta/fastq) per sample.

**Figure 3:**
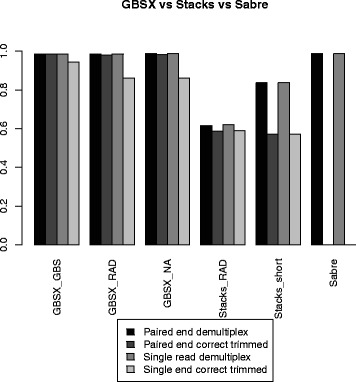
**The tools and their options comparing the percentage of demultiplexed reads and correctly trimmed reads from total reads, for paired-end and single end data.** The GBSX option NA indicates an enzyme was not provided for demultiplexing. Sabre does not perform trimming and therefor has no trimming values in the plot.

Overall, we find GBSX demultiplexing to be equal to Sabre and more sensitive than Stacks (Table 1 and Figure 3). GBSX and Sabre use a similar barcode recognition algorithm explaining their near identical results when not including the restriction enzyme recognition site. For GBSX, the demultiplexing of GBS and RAD data also use the same algorithm, as indicated by identical results. In addition, the barcode needed for the demultiplexing is always in the first read of paired-end data which means paired-end and single end read data should have identical results. This is true for GBSX and Sabre, but not Stacks. Stacks does filter reads on quality which could potentially cause discordance between single and paired-end analyses. Stacks had no misassigned barcodes, while GBSX misassigned less than one in a million reads when using a restriction enzyme recognition site and about four in a million without a restriction enzyme recognition site (due to a 4 bp shorter recognition site). Though GBSX introduces a very small number of misassigned barcodes, it does provide much greater sensitivity (GBSX >98% vs. Stacks 62/84%, Table 1) which we believe provides a more favorable demultiplexing tool.

**Table 1:** **Demultiplexing statistics as an accumulation of ten synthetic data sets, totaling 12,579,549 synthetic reads**

**Paired end reads**	**RE**	**# Misassigned**	**Misassignments**	**# Correctly**	**Sensitivity**
	**Site**		**per million reads**	**demultiplexed**	
GBSX GBS	Y	7	0.6	12,394,916	98.53%
GBSX RAD	Y	7	0.6	12,394,916	98.53%
GBSX NA	N	54	4.3	12,410,223	98.65%
Stacks process_radtags	Y	0	0.0	7,756,281	61.66%
Stacks process_shortreads	N	0	0.0	10,529,102	83.70%
Sabre	N	54	4.3	12,410,223	98.65%
**Single end reads**	**RE**	**# Misassigned**	**Misassignments**	**# Correctly**	**Sensitivity**
	**Site**		**per million reads**	**demultiplexed**	
GBSX GBS	Y	7	0.6	12,394,916	98.53%
GBSX RAD	Y	7	0.6	12,394,916	98.53%
GBSX NA	N	54	4.3	12,410,223	98.65%
Stacks process_radtags	Y	0	0.0	7,820,903	62.17%
Stacks process_shortreads	N	0	0.0	10,530,753	83.71%
Sabre	N	54	4.3	12,410,223	98.65%

NA indicates an enzyme was not provided to GBSX for demultiplexing. The RE Site column indicates if the reads did or did not contain the ApeKI restriction enzyme sequence.

Trimming was evaluated for GBSX and Stacks (Sabre does not perform trimming). Evaluation was done by counting if the length of the trimmed demultiplexed reads matched the length of the original sequences. If the trimmed sequence had a different length than the original, because it was trimmed too short or was not trimmed at all, the sequence was counted as incorrect. Overall, the GBSX GBS demultiplexed and trimmed reads performed best with the lowest errors and highest sensitivity for both paired-end and single end reads (Table 2 and Figure 3). This performance can be explained in that the GBSX GBS demultiplexer and trimmer is the only tool that uses the extra restriction enzyme recognition site before the adapter for the trimming procedure. For paired-end reads, all GBSX analyses had less errors and higher sensitivity than Stacks, likely due to the correction made possible by looking at the overlap between read pairs. The only case where equivalent data was trimmed better by Stacks was analyzing single end reads with restriction enzyme sequence for the GBSX RAD demultiplexer compared to Stacks’s process_radtags, though the GBSX GBS demultiplexer still performs better than both. The GBSX GBS performs better than GBSX RAD, mostly due to the missing of the enzyme site recognition in RAD data. In conclusion, the GBSX trimming process provides overall higher sensitivity and less errors than Stacks.

**Table 2:** **Trimming statistics on demultiplexed reads from Table** **1**

**Paired end reads**	**RE**	**# Correctly**	**Sensitivity**	**Triming errors**
	**Site**	**trimmed**		**per thousand reads**
GBSX GBS	Y	12,381,497	99.89%	1
GBSX RAD	Y	12,313,669	99.34%	7
GBSX NA	N	12,364,365	99.63%	4
Stacks process_radtags	Y	7,407,377	95.50%	45
Stacks process_shortreads	N	7,190,394	68.29%	317
**Single end reads**	**RE**	**# Correctly**	**Sensitivity**	**Triming errors**
	**Site**	**trimmed**		**per thousand reads**
GBSX GBS	Y	11,878,172	95.83%	42
GBSX RAD	Y	10,821,969	87.31%	127
GBSX NA	N	10,835,239	87.31%	127
Stacks process_radtags	Y	7,421,337	94.89%	51
Stacks process_shortreads	N	7,191,440	68.29%	317

The RE Site column indicates if the reads did or did not contain the ApeKI restriction enzyme sequence. Sabre does not perform read trimming and is therefor not included in this comparison.

## Conclusions

GBSX provides an easy to use suite of tools for designing and demultiplexing of GBS experiments. This includes evaluating restriction enzymes with the *in silico* digest tool and creating in-line barcodes with GBSX’s barcode generator. Post sequencing, GBSX provides tools to demultiplex and trim reads with, in a majority of cases, enhanced performance over existing tools. These demultiplexed and trimmed fastq files can then be mapped with traditional next generation sequencing tools and SNPs called using existing tools such as GATK [18-20] or FreeBayes [21]. In conclusion, GBSX fills in the gaps and enhances current modules for a complete GBS pipeline, from experimental design to final analyses.

## Availability and requirements

- **Poject name:** GBSX
- **Poject home page:**https://github.com/GenomicsCoreLeuven/GBSX
- **Operating system:** Platform independent
- **Programming language:** Java and Perl
- **Other requirements:** Java 1.5 or higher, BioPerl
- **License:** GNU GPL v3
- **Any restrictions to use by non-academics:** no
